# Sulfonamides in ophthalmology: adverse reactions: Evidence-based use of sulfa drugs in ophthalmology

**DOI:** 10.1007/s10792-024-03045-5

**Published:** 2024-05-03

**Authors:** David Daniel, Stephen Bacchi, Robert Casson, Weng Chan

**Affiliations:** The University of Adelaide, Adelaide, Australia

Dear Editor,

The term “sulfa drugs” may be variably conceptualised to include discrete groups such as sulfonylarylamines, non-sulfonylarylamines, sulfhydryl drugs, and sulphate drugs [1]. As such, the sulfonamide (‘sulfa’) drug class consists of a wide variety of medications, often divided into antimicrobial and non-antimicrobial drugs (see Table 1. Grouping drugs by relevant common characteristics are useful; however, the sulfa drug class is both structurally and functionally diverse. Hence, clinicians need to be careful not to extrapolate an adverse reaction (immunologically mediated or non-immunologically mediated from one sulfa drug to the entire class, which can be understood by exploring the functional structure of sulfa drugs.

**Table 1:** Examples of sulfa drugs and their use in clinical practice

Sulfonamide Antimicrobial Drug	Usage	Reported AR	Non- Antimicrobial Sulfonamide Drug	Usage	Reported AR
Sulfacetamide	Inflammatory ocular conditions and skin infections	Dryness, erythema, pruritis	Sulfonylureas	Anti-diabetic	Hypoglycaemia, weight gain
Sulfadiazine	UTIs	Eosinophilia, agranulocytosis	Sulfasalazine	DMARD	Emesis, oligospermia
Sulfamethoxazole (+ Trimethoprim)	UTIs Otitis media Bronchitis	Rash, fever, nausea	Sumatriptan Topiramate	Anti-migraine	Pain at injection site, ears, nose & throat
Sulfamethopyrazine	UTIs Malaria	Blurred vision, agranulocytosis,	Brinzolamide	Anti-glaucoma	Ocular irritation, transient blurred vision
Sulfasoxazole	Bladder infections Ear infections Meningitis	Nausea, emesis, abdominal pain	Apricoxib Celecoxib Parecoxib	NSAIDs	Nausea, dyspepsia, GI ulceration, diarrhoea
Sulfadoxine (+Pyrimethamine)	UTIs URTIs Malaria	Skin blistering, fatigue	Amprenavir Darunavir Tipranavir	Antiretrovirals	Headache, diarrhoea, abdominal pain
Sulfanilamide	Vulvovaginal candidiasis	Local irritation	Asunaprevir Beclabuvir Dasabuvir	Hepatitis C Antivirals	Nausea, insomnia, asthenia
Mafenide	Bacterial infections Fungal infections	Discolouration of skin, dark coloured urine	Acetazolamide Furosemide Hydrochlorothiazide	Diuretics	Electrolyte disturbances, dehydration

List of several antimicrobial and non-antimicrobial sulfa drugs, their use in clinical practice and some of their reported AR’s (either immunologically mediated, or non-immunologically mediated adverse reactions)

Although all sulfa drugs must contain a SO_2_NH_2_ moiety, antimicrobial and non-antimicrobial sulfa drugs maintain key structural differences. Antimicrobial sulfa drugs contain an arylamine group at the N4 position and a five or six membered ring at the N1 position, both of which are important to function and hypersensitivity reactions. Furthermore, each type of Gel Coomb hypersensitivity reaction is documented in response to sulfa drugs. Of these, Type IV reactions are more common, where sulfonamide metabolites are seemingly driving factors of these delayed reactions [2]. In comparison, there are many sulfa drug adverse reactions which are non-immune mediated, especially in the context of ophthalmology. Amongst others, adverse reactions from sulfa drugs such as thioridazine are known to cause blurred vision and dyschromatopsia [3], whilst Malagola et al. [4] described a case of retinal folds and papillary oedema due to acetazolamide.

Acetazolamide is amongst the most used sulfa drugs in ophthalmology and has multiple indications. Acetazolamide is not an antimicrobial and does not have an arylamine group. There is no clear evidence for cross reactivity between antimicrobial and non-antimicrobial groups. Therefore, patients with a sulfa allergy do not necessarily have to avoid acetazolamide [5]. After consideration of factors such as the severity of the previous self-reported reaction (e.g. not if a previous life-threatening reaction) and potential benefit to patient, cautious prescription of acetazolamide may be appropriate in individuals with a self-reported sulfa allergy, especially if the allergy pertained to an antimicrobial.

Overgeneralisation in regards to a sulfa allergy could lead to poor pharmacological choices and patient outcomes. This challenges the categorisation system of labelling people with a sulfa allergy [1]. The usage of such terminology should distinguish between a hypersensitivity rection or non-immune mediated reaction, and antimicrobial or non-antimicrobial medications. Both with respect to systemic AR and retinal disease, an argument could be made that evaluation of the likelihood of AR with these medications may be better performed on a drug-by-drug basis than by potentially confusing category terms.
